# Gut microbiota and pancreatic cancer risk, and the mediating role of immune cells and inflammatory cytokines: a Mendelian randomization study

**DOI:** 10.3389/fimmu.2024.1408770

**Published:** 2024-07-25

**Authors:** Zhiting Chen, Zhe Wang, Hejing Bao, Shudong Ma

**Affiliations:** ^1^ Department of Oncology, Nanfang Hospital, Southern Medical University, Guangzhou, China; ^2^ Department of Oncology, Panyu Central Hospital, Guangzhou, China

**Keywords:** Mendelian randomization, Gut Microbiota, Pancreatic Cancer, immune cells, inflammatory cytokines

## Abstract

**Introduction:**

Gut microbiota (GM) influences the occurrence and development of pancreatic cancer (PC), potentially through the involvement of inflammatory cytokines (IC) and immune cells (IM). We aimed to investigate the causal impact of the gut microbiota (GM) on pancreatic cancer (PC) and identify potential IC and IM mediators.

**Methods:**

The summary statistics data from whole-genome association studies of gut microbiota, immune cells, inflammatory cytokines, and four types of pancreatic tumors (MNP: Malignant neoplasm of pancreas; BNP: Benign neoplasm of pancreas; ADCP: Adenocarcinoma and ductal carcinoma of pancreas; NTCP: Neuroendocrine tumor and carcinoma of pancreas). Two-sample univariable Mendelian randomization (UVMR), multivariable Mendelian randomization (MVMR), and mediation analysis were employed to assess the causal relationship between gut microbiota (GM) and pancreatic cancer (PC), as well as potential IC and IM mediators.

**Results:**

The two-sample UVMR analysis showed causal relationships between 20 gut microbiota species and pancreatic cancer, with pancreatic cancer affecting the abundance of 37 gut microbiota species. Mediation analysis revealed that Interleukin-6 (IL-6), “CD4 on naive CD4+ T cell” and “SSC-A on HLA DR+ Natural Killer” mediated the causal effects of gut microbiota on pancreatic cancer.

**Conclusion:**

This Mendelian randomization study demonstrates causal relationships between several specific gut microbiota and pancreatic cancer, as well as potential mediators (IC, IM).

## 1 Introduction

Pancreatic cancer (PC) ranks among the deadliest malignancies in humans, with its mortality closely tied to its incidence (1). Extensive studies suggest that gut microbiota (GM) may play a potential role in the occurrence of PC or modulating individual responses to tumor treatment. Specific mechanisms include inflammatory responses, immune system regulation, metabolic influences, and alteration of the tumor microenvironment (2).

Inflammation, as a defensive response of the body to harmful stimuli, involves the regulation and activation of immune cells and inflammatory cytokines. In the pathogenesis of microbiota-related PC, inflammation is considered a primary driving factor (2). Inflammatory diseases such as chronic pancreatitis are recognized risk factors for PC (3). Although the potential sources of infection for PC remain unclear to date, microbial infections from the intestine are believed to be the main trigger for inflammation. Gut microbiota triggers inflammatory responses, promotes the secretion of inflammatory cells and factors, enhances exposure to oxidative stress, leading to molecular changes and transformation, thereby promoting tumor development (4). Furthermore, inflammatory responses are often accompanied by immune reactions. The activation of the innate immune system may be a key factor in promoting PC occurrence. Previous studies have shown that after the destruction of the intestinal mucosa, specific intestinal microbes can enter lymph nodes and spleen, activating specific immune cells (5, 6). Moreover, specific microbes in the intestine can establish an immunosuppressive tumor microenvironment in PC, promoting cancer progression and resistance to immune therapy (7). These studies indicate the association of gut microbiota, inflammatory responses, immune cells with the occurrence and development of PC, which can also affect treatment outcomes. The role and mechanisms of gut microbiota in PC occurrence require closer attention. Therefore, we aim to clarify these relationships and identify potential gut microbiota and targets for early diagnosis and clinical treatment.

Mendelian randomization (MR) is a method that uses genetic variation as instrumental variables (IVs) to infer causal relationships between exposure and clinical outcomes. It can control potential confounding factors and avoid reverse causation bias (8). Additionally, an increasing number of Genome wide association studies (GWAS) studies have identified human genetic information related to gut microbiota (9). Therefore, we employ MR to infer the causal relationship between gut microbiota and PC, further dissecting the associations among gut microbiota, inflammatory responses, immune cells, and PC.

## 2 Materials and methods

### 2.1 Study design

**Figure 1:**
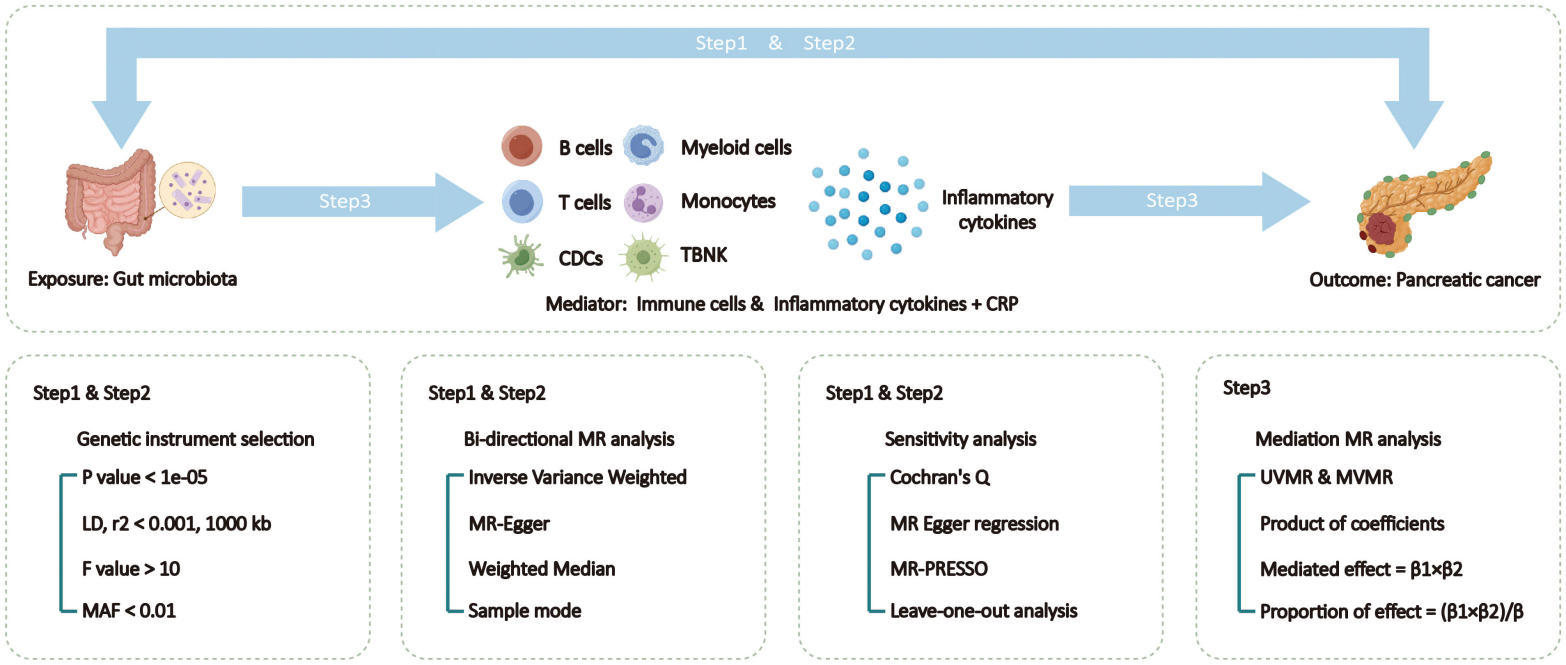
Overview of the Mendelian Randomization (MR) study design. MAF, minor allele frequency; MR, Mendelian randomization; MVMR, multivariable Mendelian randomization; TSMR, two sample Mendelian randomization; TBNK, T cells B cells and natural killer cells.

The specific design and process of this MR study are divided into 3 steps (**Figure 1**). In the first and second steps, we utilized bi-directional two-sample univariable Mendelian randomization (UVMR) to assess the causal relationships between exposure and outcome, following the three main assumptions of MR analysis: selected SNPs (1) should be closely associated with exposure (2), affect the outcome only through exposure, and (3) should not be associated with potential confounding factors (10). Reverse analysis reveals results with reverse causal relationships. And validate the results of UVMR using external data. In the third step, we employed both UVMR and multivariable Mendelian randomization (MVMR) to analyze the mediation effects of mediators between exposure and outcome, calculating the effect values and proportions for each qualified mediator. This study adhered to the Strengthening the Reporting of Observational Studies in Epidemiology using Mendelian Randomization (STRBOE-MR) guidelines (**Supplementary Table 1**) (11).

### 2.2 Data sources

This study use gut microbiota GWAS data from the Dutch Microbiome Project (DMP) as exposures. This study included 7,738 people of European descent (12). The data was determined through shotgun metagenomic sequencing of fecal samples, encompassing a total of 207 taxonomic units (5 phyla, 10 classes, 13 orders, 26 families, 48 genera, and 105 species).

This study conducted mediation analysis using GWAS summary data of immune cell phenotypes and inflammatory cytokines. The immune cell phenotype data included 3,757 individuals of European ancestry from non-overlapping cohorts, comprising 731 immune features: absolute cell counts (AC, n=118), median fluorescence intensity reflecting surface antigen levels (MFI and SAL, n=389), morphological parameters (MP, n=32), and relative cell counts (RC, n=192) (13). These features, such as MFI, AC, and RC, include mature stages of B cells, CDCs, T cells, monocytes, myeloid cells, TBNK (T cells, B cells, and natural killer cells), and Treg panels. The MP features encompass CDC and TBNK panels. Inflammatory cytokines comprised 41 inflammation-modulating cytokines and CRP. Data for the 41 systemic inflammation-modulating cytokines were obtained from meta-analyses of cytokine-related GWAS from three independent population cohorts, including 8,293 Finnish individuals from the Young Finns Study and the FINRISK studies (FINRISK1997 and FINRISK2002) (14). These 41 cytokines were initially normalized through a first-step inverse transformation of cytokine distributions, followed by a second-step inverse transformation of residuals from linear regression models of transformed cytokines on age, sex, body mass index (BMI), and genetic principal components. CRP data originated from a meta-analysis of GWAS involving 158 European individuals from the Cytokine Working Group (CIWG) consortium, with adjustments made for age, sex, and population structure for 15 genetic associations between 2.4 million genetic variants and log-transformed CRP levels (15).

This study utilized GWAS summary statistics data from the FinnGen Consortium R10 release for four types of pancreatic tumors as outcomes, diagnosis was made using International Classification of Diseases, 10th Revision (ICD-10) diagnosis codes (16). These included Malignant neoplasm of pancreas (MNP, cases=1626, controls=312567) and Benign neoplasm of pancreas (BNP, cases=603, controls=410975), as well as two pathological subtypes: Adenocarcinoma and ductal carcinoma of pancreas (ADCP, cases=731, controls=313462) and Neuroendocrine tumor and carcinoma of pancreas (NTCP, cases=129, controls=314064). Validation of UVMR results External pancreatic cancer data from GWAS Catalog (cases=1196, controls=475049) (17). Please refer to **Table 1** for specific details of all the data.

**Table 1:** Data sources. DMP, Dutch Microbiome Project.

Phenotypes	Cases/controls or samplesizes	Data source	Phenotypic code	Ancestry
Exposure				
Gut microbiota	7,738	DMP	GCST90027446 to GCST90027857	European
Mediator				
Immune cells	3,757	Orrù V. et al.	GCST0001391 to GCST0002121	European
Inflammatory cytokines	8,293	Ahola-Olli et al.	NA	European
C-reactive protein	575,531	Said et al.	GCST90029070	European
Outcome				
ADCP	731/313,462	FinnGen	C3_PANCREAS_ADENO_DUCTAL_EXALLC	European
BNP	603/410,975	FinnGen	CD2_BENIGN_PANCREAS	European
MNP	1,626/312,567	FinnGen	C3_PANCREAS_EXALLC	European
NTCP	129/314,064	FinnGen	C3_PANCREAS_NEUROENDOCRINE_EXALLC	European
PC	1,196/476,245	GWAS Catalog	ebi-a-GCST90018893	European

NA, No available phenotypic code.

### 2.3 Genetic instrumental variable selection

We employed the following steps to select instrumental variables (IVs) for analysis. Firstly, to obtain more effective SNPs, the significance threshold for IVs of GM, IC, and IF was set at 1e-05, while for the IVs of the four pancreatic tumors, it was set at 1e-06. Secondly, PLINK software (version v1.90) was used to remove SNPs with linkage disequilibrium (LD) r2 <0.001 within a distance of 10,000 kb (18). Thirdly, SNPs significantly associated with the outcomes (with a significance threshold of 5e-05) were excluded. Fourthly, palindromic SNPs were removed to ensure that the effect of SNPs on exposure and outcome corresponded to the same allele. Finally, we calculated the F-statistic values to measure the strength of IVs (19), retaining SNPs with F-values greater than 10 and excluding those with a minimum allele frequency (MAF) less than 0.01. Detailed information regarding these IVs can be found in **Supplementary Table 2**.

### 2.4 UVMR and MVMR analysis

In assessing the causal relationship between GM and the four types of pancreatic tumors, the Inverse Variance Weighted (IVW) method was employed as the primary approach (8), with four other methods used for supplementary analysis (Weighted Median, MR-Egger, Weighted Mode, and Sample mode). When reporting results for binary outcomes, odds ratios (ORs) were presented along with 95% confidence intervals, and when the results were for continuous variables, β values were reported. In the MVMR analysis, the Multivariable Inverse Variance Weighted (MV-IVW) method was used as the primary analysis. Results with a p-value <0.05 were considered significant. Correction for multiple testing of all IVW results was performed using the False Discovery Rate (FDR) method, and FDR q-values were provided. The same approach was used for external validation.

### 2.5 Mediation MR analysis

**Figure 2:**
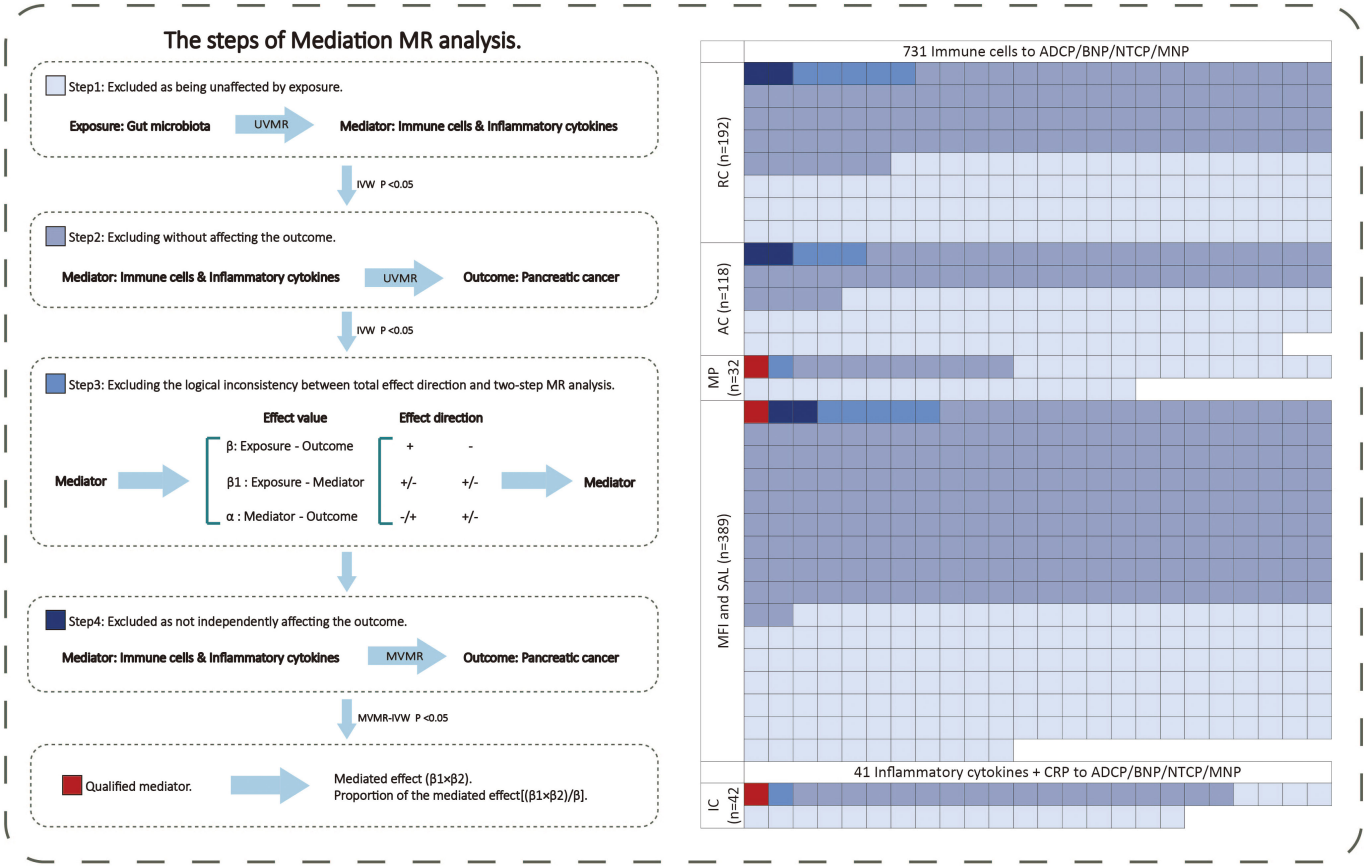
The mediating selection process in the causal relationship between the exposure (gut microbiota) and outcome (pancreatic cancer). AC, absolute cell counts; ADCP, Adenocarcinoma and ductal carcinoma of pancreas; BNP, Benign neoplasm of pancreas; CRP, C-reactive protein; GM, gut microbiota; IC, inflammatory cytokines; MFI, median fluorescence intensity; MNP, Malignant neoplasm of pancreas; MP, morphological parameters; MR, Mendelian randomization; MVMR, multivariable Mendelian randomization; NTCP, Neuroendocrine tumor and carcinoma of pancreas; RC, relative cell counts; SAL, surface antigen levels; UVMR, univariable Mendelian randomization.

We conducted the screening of potential mediators in the gut microbiota-pancreatic tumor pathway through the following steps (**Figure 2**). In the first step, the UVMR was used to select the mediators influenced by exposure causally, and their effect values (β1) were calculated. In the second step, the UVMR was used to select the mediators identified in the first step that causally affected the outcome, and their effect values (α) were calculated. In the third step, based on the direction of the obtained exposure-outcome (β), exposure-mediator (β1), and mediator-outcome (α) effect sizes, mediators consistent with logic were retained (if the total effect of exposure on the outcome β is positive, then both β1 and α should be positive or negative; conversely, if the total effect of exposure on the outcome β is negative, then β1 and α should be one positive and one negative). In the fourth step, the MVMR was used to assess the causal effects of mediators on the outcome after adjusting for exposure effects. The mediator with MV-IVW P-value <0.05 is regarded as the final result. Subsequently, combining the causal effect of exposure on the outcome obtained from UVMR (β), the “product of coefficients” method was used to calculate the mediated effect values of mediators in the gut microbiota-pancreatic tumor pathway (β1 × β2) and the proportion of the effect values ([β1 × β2]/β) (20).

### 2.6 MR sensitivity analysis

We conducted sensitivity analyses using the MR Egger regression method, leave-one-out analysis, and MR-PRESSO method. Cochran’s Q statistic was computed for each SNP to assess heterogeneity, and the p-value from the MR Egger regression intercept test was used to evaluate horizontal pleiotropy (21). The MR-PRESSO method was employed to correct for potential horizontal pleiotropy in the selected IVs (22). P_heterogeneity <0.05 was considered indicative of heterogeneity, while P_Global.test and P_pleiotropy <0.05 were considered indicative of pleiotropy. Evidence of pleiotropy would suggest lack of causal evidence.

All analyses were performed using the TwoSampleMR (23), MR-PRESSO (22), and MendelianRandomization (24) packages in R software (version 4.3.0).

## 3 Results

### 3.1 Genetic instruments for exposures

Through the above steps, the number of SNPs for GM ranged from 4 to 60 (median=28), for immune cells ranged from 10 to 189 (median=24), for inflammatory cytokines ranged from 6 to 30 (median=18), and for pancreatic tumors ranged from 201 to 855 (median=622). Additionally, all SNPs had an F-statistic greater than 15, indicating the absence of weak instrumental variables (**Supplementary Table 2**).

### 3.2 Causal associations of gut microbiota with pancreatic cancer

**Figure 3:**
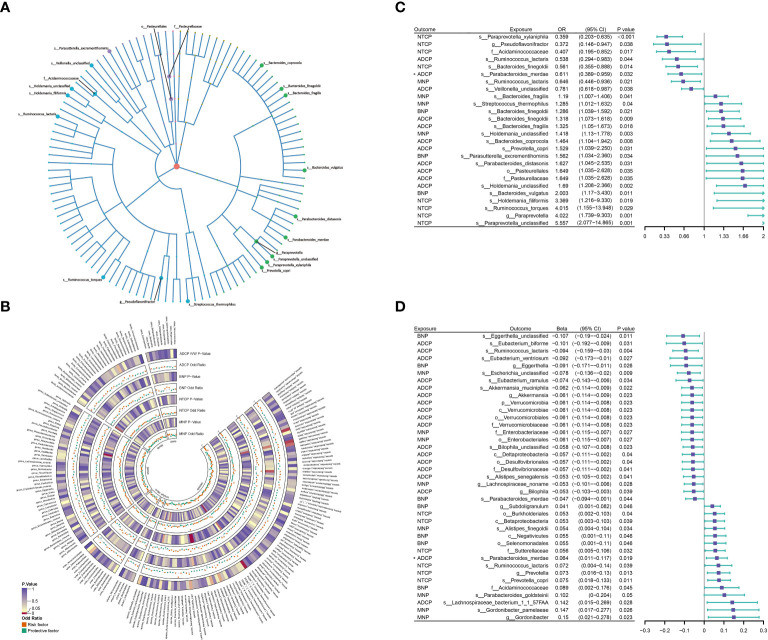
**(A)** 21 varieties of gut microbiota are causal related to four different types of pancreatic cancer. **(B)** IVW method results of MR analysis between GM and PC. **(C, D)** Results of Bi-directional Univariate Mendelian Randomization on the interplay between the gut microbiota and pancreatic cancer. ADCP, Adenocarcinoma and ductal carcinoma of pancreas; BNP, Benign neoplasm of pancreas; GM, gut microbiota; MNP, Malignant neoplasm of pancreas; NTCP, Neuroendocrine tumor and carcinoma of pancreas. The prefix “c_/o_/f_/g_/s_” represents class/order/family/genus/species respectively. The “*” symbol represents a reverse causal relationship.

Among the 207 types of gut microbiota included in the analysis, a total of 20 gut microbiota (representing 1 order, 2 families, 2 genera, and 15 species from p_Bacteroidetes, p_Firmicutes, and p_Proteobacteria) were found to have a causal relationship with outcome (**Figure 3A**). **Figure 3B** presents the results of evaluating the causal effects of gut microbiota on the four types of pancreatic tumors using the IVW method as the primary approach. When ADCP was considered as the outcome, it was observed that s_Ruminococcus_lactaris (OR=0.538; P=0.042) and s_Veillonella_unclassified (OR=0.781; P=0.038) were inversely associated with its risk, while s_Bacteroides_finegoldii (OR=1.318; P=0.009), s_Bacteroides_fragilis (OR=1.325; P=0.018), s_Bacteroides_coprocola (OR=1.464; P=0.008), s_Prevotella_copri (OR=1.529; P=0.031), s_Parabacteroides_distasonis (OR=1.627; P=0.031), o_Pasteurellales (OR=1.649; P=0.035), f_Pasteurellaceae (OR=1.649; P=0.035), and s_Holdemania_unclassified (OR=1.690; P=0.002) were positively associated with its risk. When BNP was considered as the outcome, it was found that s_Bacteroides_finegoldii (OR=1.286; P=0.021), s_Parasutterella_excrementihominis (OR=1.562; P=0.034), and s_Bacteroides_vulgatus (OR=2.003; P=0.011) were positively associated with its risk. When MNP was considered as the outcome, it was found that s_Ruminococcus_lactaris (OR=0.646; P=0.021) was inversely associated with its risk, while s_Bacteroides_ fragilis (OR=1.190; P=0.041), s_Streptococcus _thermophilus (OR=1.285; P=0.040), and s_Holdemania_unclassified (OR=1.418; P=0.003) were positively associated with its risk. When NTCP was considered as the outcome, it was found that s_Paraprevotella_ xylaniphila (OR=0.359; P=0.0004), g_Pseudoflavonifractor (OR=0.372; P=0.038), f_Acidaminococcaceae (OR=0.407; P=0.017), and s_Bacteroides_finegoldii (OR=0.561; P=0.014) were inversely associated with its risk, while s_Holdemania_filiformis (OR=3.369; P=0.019), s_Ruminococcus_torques (OR=4.015; P=0.029), g_Paraprevotella (OR=4.022; P=0.001), and s_Paraprevotella_ unclassified (OR=5.557; P=0.0006) were positively associated with its risk (**Figure 3C**; **Supplementary Table 3**). In addition, we conducted external validation on the gut microbiota that has a causal relationship with pancreatic tumors, Using IVW as the main analysis method, the validation results of the other gut microbiota, except for s_Bacteroidees_vulgatus, s_Prevotella_copri, s_Holdemania_filiformis, and f_Acidamococcaceae, are consistent with the findings. The results are presented in **Supplementary Table 3**.

When assessing the reverse causal effects of pancreatic tumors on gut microbiota, using the IVW method as the primary approach, it was found that the abundance of 37 gut microbiota (including 1 phylum, 4 classes, 5 orders, 5 families, 7 genera, and 15 species from p_Actinobacteria, p_Bacteroidetes, p_Firmicutes, p_Proteobacteria, and p_Verrucomicrobia) could be influenced by four types of pancreatic tumors. Specifically, when ADCP was considered as the exposure, it was observed that the abundance of s_Parabacteroides_merdae (β=0.064; P=0.019) could be influenced, showing a contradictory positive causal effect, which violates the fundamental assumption of MR analysis. Therefore, s_Parabacteroides_merdae was not considered to have an effect on ADCP (**Figure 3D**; **Supplementary Table 3**).

Sensitivity analyses were conducted on the results of the above UVMR analyses. All results passed heterogeneity analysis and pleiotropy analysis, with P-values from heterogeneity analysis, MR-Egger intercept, and MR-PRESSO all greater than 0.05, indicating the absence of minimal heterogeneity and horizontal pleiotropy (**Supplementary Table 8**). Additionally, Leave-one-out analysis showed that in the bidirectional MR analysis, no single SNP significantly altered the causal effects (**Supplementary Figures 1**, **2**).

### 3.3 Mediation analyses of potential mediators

**Figure 4:**
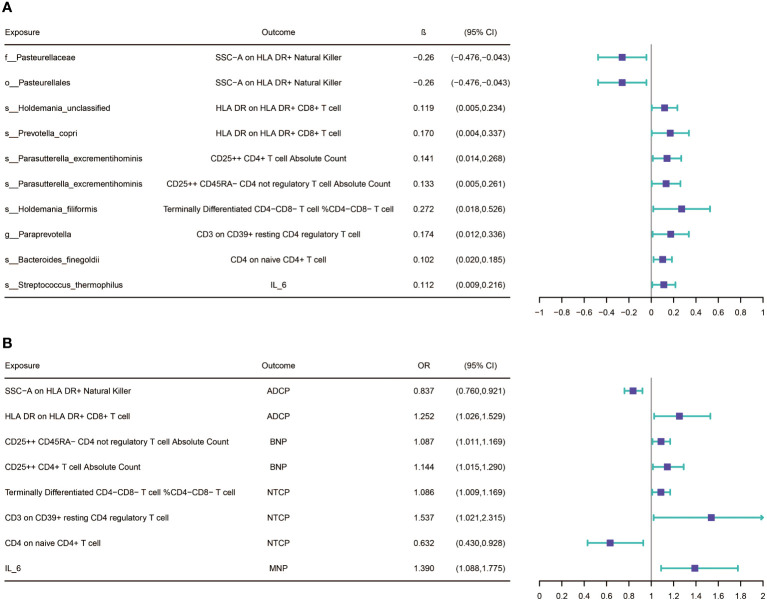
**(A)** Results of Bi-directional Univariate Mendelian Randomization on the interplay between the gut microbiota and mediators(immune cells and inflammatory cytokines. **(B)** Results of Bi-directional Univariate Mendelian Randomization on the interplay between the mediators and pancreatic cancer. ADCP, Adenocarcinoma and ductal carcinoma of pancreas; BNP, Benign neoplasm of pancreas; MNP, Malignant neoplasm of pancreas; NTCP, Neuroendocrine tumor and carcinoma of pancreas. The prefix “c_/o_/f_/g_/s_” represents class/order/family/genus/species respectively.

**Figure 5:**
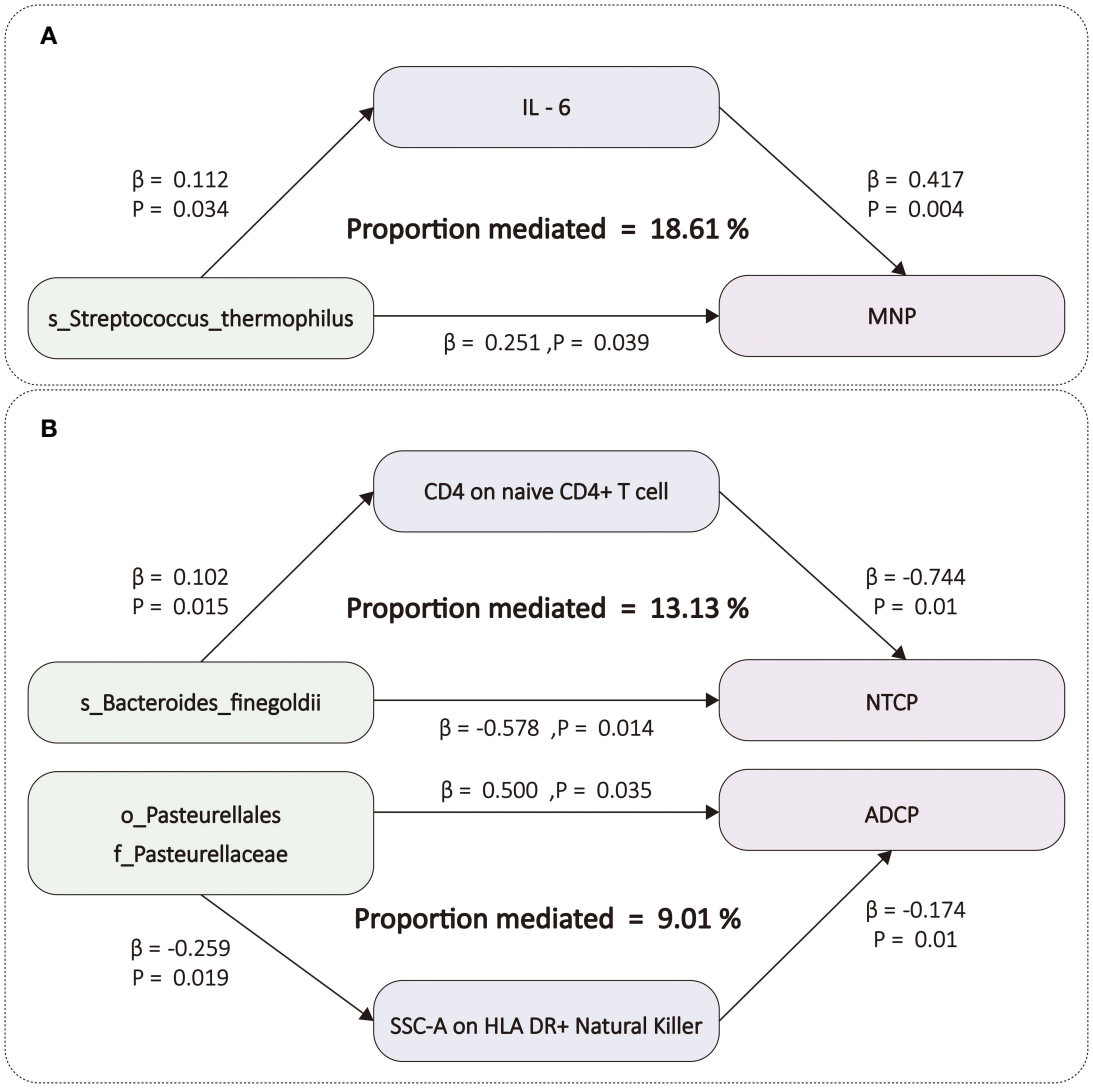
**(A)** The figure shows the mediation mode of “GM–IC–PC. **(B)** The figure shows the mediation mode of “GM–IM–PC”. ADCP, Adenocarcinoma and ductal carcinoma of pancreas; GM, gut microbiota; IC, inflammatory cytokines; IM, immune cells; IL-6, Interleukin-6; MNP, Malignant neoplasm of pancreas; NTCP, Neuroendocrine tumor and carcinoma of pancreas. The prefix “c_/o_/f_/g_/s_” represents class/order/family/genus/species respectively.

After screening for potential intermediate factors, we identified a total of 1 inflammatory cytokine and 2 immune cell phenotypes that met our selection criteria. Initially, through two-step UVMR and directional screening based on β, β1, and α, we preliminarily selected 9 gut microbiota-immune cell-pancreatic tumor pathways and 1 gut microbiota-inflammatory cytokines-pancreatic tumor pathway, which included 7 immune cell phenotypes and 1 inflammatory cytokine (**Figure 4**; **Supplementary Tables 4**-**7**). Subsequently, in the fourth step, we used MVMR to evaluate whether the selected intermediates could independently influence the outcomes and calculated the mediation effect values and proportions of the effect values. After adjusting for the influence of gut microbiota, it was found that “SSC-A on HLA DR+ Natural Killer” remained significantly associated with ADCP, “CD4 on naive CD4+ T cell” remained associated with NTCP, and IL-6 remained associated with MNP. Finally, we found that “SSC-A on HLA DR+ Natural Killer” mediated the causal associations between o_Pasteurellales and f_Pasteurellaceae with ADCP, with a mediation proportion of 9.01% (P=0.01), “CD4 on naive CD4+ T cell” mediated the causal association between s_Bacteroides_finegoldii and NTCP, with a mediation proportion of 13.13% (P=0.01), and IL-6 mediated the causal association between s_Streptococcus_thermophilus and MNP, with a mediation proportion of 18.61% (P=0.004) (**Figure 5**; **Table 2**).

**Table 2:** MVMR estimates for the causal associations of mediators with outcomes with adjustment for exposure.

Oucome	Mediator	OR	(95%CI)	IVW P value	Adjust for	β	(95%CI)	MV-IVW P value
Mediator: Immune cells								
UVMR analysis					MVMR analysis			
ADCP	SSC-A on HLA DR+ Natural Killer	0.837	(0.760, 0.921)	0.0002	o_Pasteurellales	-0.174	(-0.306, -0.042)	0.010
ADCP	SSC-A on HLA DR+ Natural Killer	0.837	(0.760, 0.921)	0.0002	f_Pasteurellaceae	-0.175	(-0.306, -0.043)	0.010
ADCP	HLA DR on HLA DR+ CD8+ T cell	1.253	(1.026, 1.529)	0.027	s_Holdemania_unclassified	0.087	(-0.133, 0.306)	0.440
ADCP	HLA DR on HLA DR+ CD8+ T cell	1.253	(1.026, 1.529)	0.027	s_Prevotella_copri	0.149	(-0.065, 0.364)	0.172
BNP	CD25++ CD45RA- CD4 not regulatory T cell Absolute Count	1.087	(1.011, 1.169)	0.024	s_Parasutterella_excrementihominis	0.066	(-0.144, 0.276)	0.538
BNP	CD25++ CD4+ T cell Absolute Count	1.144	(1.015, 1.290)	0.027	s_Parasutterella_excrementihominis	0.105	(-0.086, 0.295)	0.281
NTCP	Terminally Differentiated CD4-CD8- T cell %CD4-CD8- T cell	1.086	(1.009, 1.169)	0.028	s_Holdemania_filiformis	0.072	(-0.375, 0.501)	0.741
NTCP	CD3 on CD39+ resting CD4 regulatory T cell	1.537	(1.021, 2.315)	0.040	g_Paraprevotella	0.411	(-0.166, 0.988)	0.163
NTCP	CD4 on naive CD4+ T cell	0.632	(0.430, 0.928)	0.019	s_Bacteroides_finegoldii	-0.744	(-1.307, -0.181)	0.010
Mediator: Inflammatory cytokines								
UVMR analysis					MVMR analysis			
MNP	IL_6	1.390	(1.088, 1.775)	0.008	s_Streptococcus_thermophilus	0.417	(0.134, 0.699)	0.004

Sensitive analysis was conducted on the UVMR results of the above intermediate analysis. In the analysis of gut microbiota-immune cell pathways, three causal relationships with horizontal pleiotropy were detected and subsequently excluded from our final results. However, the MR-Egger intercept and the P-value of MR-PRESSO for other analysis results were both greater than 0.05, indicating the absence of minimal horizontal pleiotropy (**Supplementary Table 8**). Heterogeneity analysis revealed potential heterogeneity in some IVs, but the instrument validity test showed that the strength of these IVs was sufficient (all F-statistics ≥ 15; **Supplementary Table 2**), and no horizontal pleiotropy was detected (all P_Global.test and P_pleiotropy > 0.05; **Supplementary Table 8**). Overall, sensitivity analysis confirmed the reliability of these results.

## 4 Discussion

In this large-scale MR study, we identified a total of 20 gut microbiota taxa (9 from p_Bacteroidetes, 8 from p_Firmicutes, 3 from p_Proteobacteria) showing genetically predisposed causal relationships with the four types of pancreatic tumors. Similarly, the four types of pancreatic tumors also exhibited genetically predisposed causal effects on 36 gut microbiota taxa (4 from p_Actinobacteria, 5 from p_Bacteroidetes, 10 from p_Firmicutes, 11 from p_Proteobacteria, 6 from p_Verrucomicrobia). Furthermore, employing mediation analysis approach using UVMR and MVMR, we identified four microbial taxa that mediate the effects on PC through one inflammatory cytokine and two immune cell phenotypes (derived from Maturation stages of T cell panel and TBNK panel).

The gut microbiota, often referred to as the “second endocrine organ,” influences the metabolism and physiological processes of the host through the production of metabolites and specific small molecules (25). In comparison to healthy individuals, patients with pancreatic cancer (PC) exhibit various microbial changes in the gastrointestinal tract (26). Analysis of microbial characteristics in human pancreatic tumors detected thirteen different phyla, with p_Proteobacteria (45%), p_Bacteroidetes (31%), and p_Firmicutes (22%) being the most abundant (7). The gut microbiota identified in our study to have causal effects on pancreatic tumors also primarily belong to these three phyla. Considering the anatomical connection between the pancreas and the digestive tract via the pancreatic duct, the characteristic microbiota found in pancreatic tumors may originate from ectopic colonization of the gut microbiota. Infections of the gut microbiota have complex associations with cancer-related inflammatory states. Gut microbiota entering the pancreas through pancreatic duct reflux may disrupt the normal microenvironment of the pancreas (2). Binding of gut microbiota to Toll-like receptors (TLRs) on the surface of local cells generates lipopolysaccharides (LPS), activating inflammatory responses, increasing recruitment of inflammatory cells and cytokine secretion, promoting the expression of CXC receptor 2 (CXCR2), CXC ligands (CXCLs), STAT3, and IL-6, activating NF-kB, leading to oxidative stress imbalance in the microenvironment, DNA damage, and ultimately tumor formation (27). Furthermore, LPS-driven inflammation can activate K-ras mutations in PC, which are present in 90% of PC cases (28). These indicate the inflammatory response caused by gut microbiota infection and the key role of IL-6 in the development of PC. Our analysis found that s_Streptococcus_thermophilus from p_Firmicutes may promote PC, especially MNP, through the inflammatory cytokine IL-6. This provides evidence for s_Streptococcus_thermophilus or IL-6 as potential targets for early diagnosis and clinical treatment of PC. The gut microbiota modulates both the innate and adaptive immune systems of their hosts, including systemic immunity and the function of the epithelial barrier they inhabit (29, 30). Additionally, they generate various antigens and metabolites that influence T cell development and immune system maturation (31). Alterations in the tumor microenvironment (TME) by gut microbiota can either promote tumor initiation or suppress tumor progression. On one hand, in murine models, antibiotic treatment targeting the gut microbiota alters the immune phenotype of TME, inducing activation of anti-tumor T cells to restrain tumor growth (32), evidenced by enhanced differentiation of CD4+ T cells into helper T cell 1 (Th1) and heightened activity of CD8+ T cells (33). On the other hand, microbes in pancreatic cancer (PC) activate selective Toll-like receptors (TLRs) in monocytes, leading to immune tolerance. TLRs, part of the pattern recognition receptor (PRR) family, orchestrate immune responses to microbial infections and accelerate tumor initiation through both innate and adaptive immunity in PC (34). Furthermore, animal studies suggest that TLR activation can induce pancreatic inflammation and synergize with K-ras to promote PC (28). Our study reveals that s_Bacteroides_finegoldii from p_Bacteroidetes and o_Pasteurellales together with f_Pasteurellaceae from p_Proteobacteria exert inhibitory and promotional effects on PC, respectively, through immune cell phenotypes “CD4 on naïve CD4+ T cell” and “SSC-A on HLA DR+ Natural Killer.” The immune phenotype “CD4 on naïve CD4+ T cell” belongs to the Maturation stages of T cell panel, indicating that s_Bacteroides_finegoldii may affect the maturation process of the aforementioned anti-tumor T cells, thereby inhibiting tumor development. Conversely, o_Pasteurellales and f_Pasteurellaceae may influence NK cells, potentially promoting tumor development through inflammatory responses. However, specific mechanisms require further exploration.

Strengths of this study include the utilization of large-scale GWAS data encompassing gut microbiota, inflammatory cytokines, immune cells, and four types of pancreatic tumors. This extensive dataset ensures robust statistical power and yields a wealth of results. Moreover, the study employs a rigorously designed analytical framework to investigate the causal relationships between gut microbiota and the four pancreatic diseases. Utilizing methods such as UVMR and MVMR, the study identifies one inflammatory cytokine and two immune cell phenotypes as potential mediators of the gut microbiota’s influence on pancreatic cancer. Finally, the study employs various MR analysis methods for causal inference and conducts sensitivity analyses to ensure the robustness of the findings, mitigating the influence of horizontal pleiotropy and other factors.

Limitations of this study include, firstly, the assumption of linearity in the causal relationship between gut microbiota and pancreatic cancer (PC) through UVMR and MVMR analyses. However, in reality, this relationship may be more intricate, involving environmental factors and other genetic determinants. Secondly, despite identifying potential mediators of the causal relationship between gut microbiota and PC, considering the complex biological processes involved in this pathway, our study may not encompass all possible mediation pathways. In addition, due to data limitations, there may be some overlap and intersection between the four pancreatic tumors data used as exposure studies, and the study population could not be defined with respect to grade and stage of pancreatic tumors. Due to the particularity of GWAS studies, there is also a lack of covariate adjustment for the data source cohort. Lastly, our study population predominantly consists of individuals of European ancestry, which may limit the generalizability of the findings.

## 5 Conclusion

In summary, the gut microbiota plays a crucial role in the occurrence and progression of PC. This study comprehensively assessed the associations between gut microbiota, inflammatory cytokines, immune cells, and PC, identifying biomarkers that could be used for predicting PC prognosis and risk. It underscores and elucidates potential mechanisms, providing new insights for targeted interventions for PC based on gut microbiota.

## Data availability statement

Publicly available datasets were analyzed in this study. This data can be found here: https://www.ebi.ac.uk/gwas/home.

## Ethics statement

The data used in our MR analysis were obtained entirely from previously reported summary data. Therefore, neither patient consent nor ethical approval were necessary for this study.

## Author contributions

ZC: Conceptualization, Formal analysis, Resources, Software, Visualization, Writing – original draft, Writing – review & editing. ZW: Conceptualization, Formal analysis, Resources, Software, Visualization, Writing – original draft, Writing – review & editing. HB: Data curation, Investigation, Methodology, Validation, Writing – original draft. SM: Funding acquisition, Project administration, Supervision, Writing – review & editing.

## Supplementary material

Supplementary Table 1 Details of the instrumental variables (IV) included in the analysis, all MR analyses, and the results of the sensitivity analyses are incorporated.

Supplementary Figure 1 Leave-one-out analysis plots of significant associations between gut microbiota and pancreatic cancer.

Supplementary Figure 2 Leave-one-out analysis plots of significant associations between pancreatic cancer and gut microbiota.
