# Supplementary figures and images for: Gut microbiota and pancreatic cancer risk, and the mediating role of immune cells and inflammatory cytokines: a Mendelian randomization study
Source: Front Immunol. 2024 Jul 25;15:1408770. doi: 10.3389/fimmu.2024.1408770 (PMC11306078; doi:10.3389/fimmu.2024.1408770)

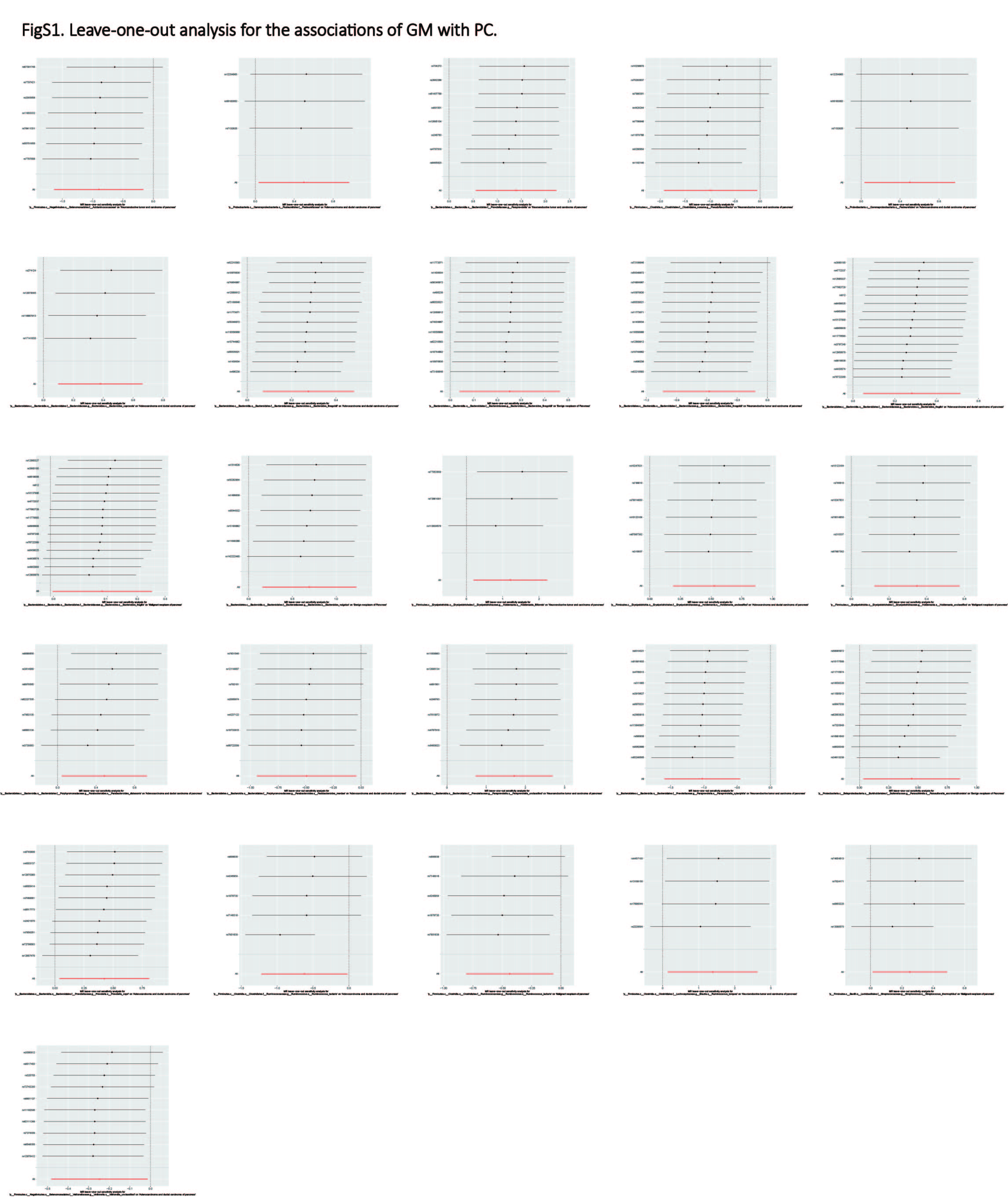

Supplement: Supplementary Figure 1 — Leave-one-out analysis plots of significant associations between gut microbiota and pancreatic cancer. [file Image_1.jpeg]

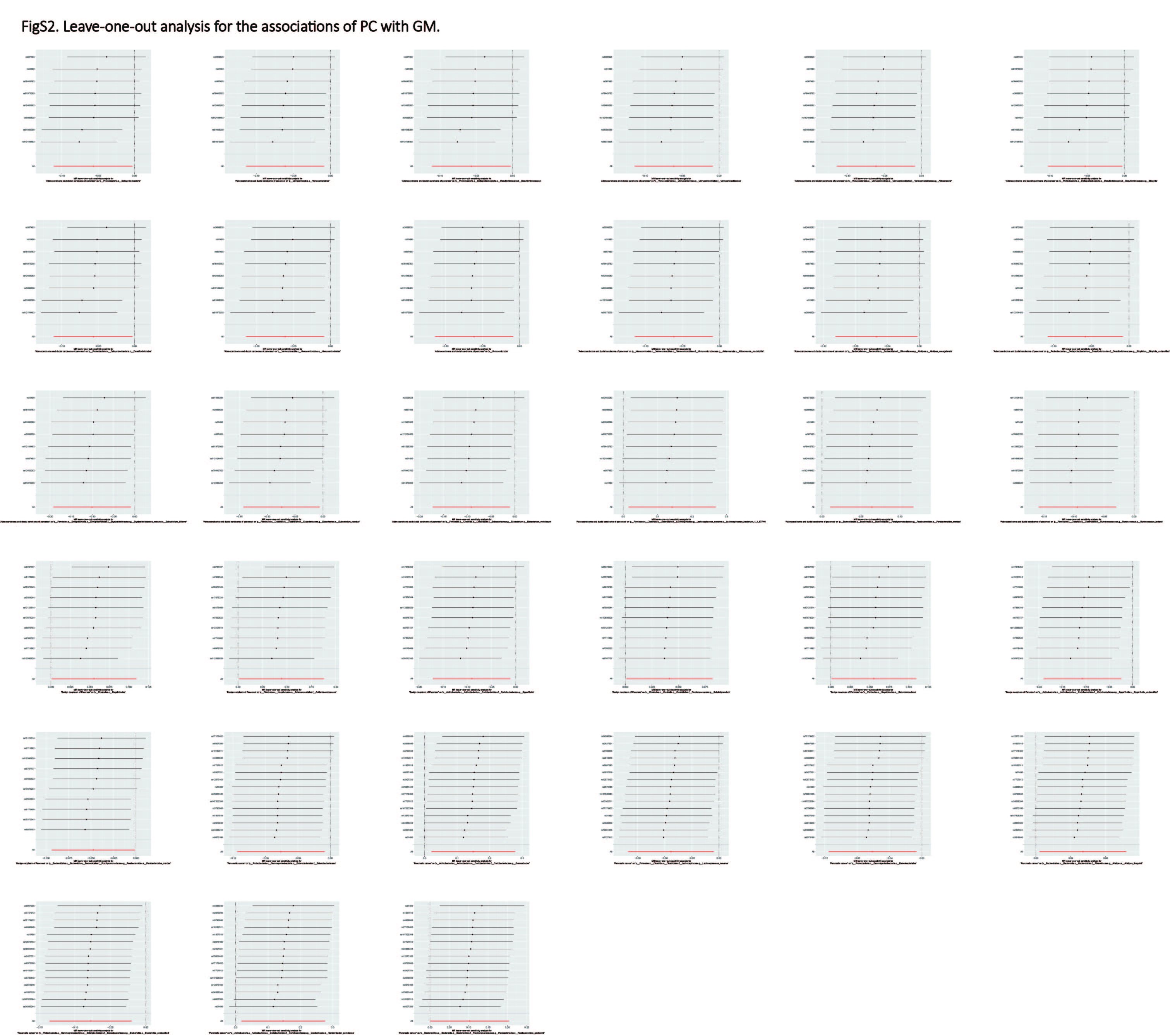

Supplement: Supplementary Figure 2 — Leave-one-out analysis plots of significant associations between pancreatic cancer and gut microbiota. [file Image_2.jpeg]
